# Tumor-infiltrating lymphocytes for treatment of solid tumors: It takes two to tango?

**DOI:** 10.3389/fimmu.2022.1018962

**Published:** 2022-10-28

**Authors:** Mohammad Hossein Kazemi, Maryam Sadri, Alireza Najafi, Ali Rahimi, Zeinab Baghernejadan, Hossein Khorramdelazad, Reza Falak

**Affiliations:** ^1^ Department of Immunology, School of Medicine, Iran University of Medical Sciences, Tehran, Iran; ^2^ Immunology Research Center, Institute of Immunology and Infectious Diseases, Iran University of Medical Sciences, Tehran, Iran

**Keywords:** combination therapy, immunotherapy, solid tumor, tumor-infiltrating lymphocyte (TIL), TIL therapy

## Abstract

Tumor-infiltrating lymphocytes (TILs), frontline soldiers of the adaptive immune system, are recruited into the tumor site to fight against tumors. However, their small number and reduced activity limit their ability to overcome the tumor. Enhancement of TILs number and activity against tumors has been of interest for a long time. A lack of knowledge about the tumor microenvironment (TME) has limited success in primary TIL therapies. Although the advent of engineered T cells has revolutionized the immunotherapy methods of hematologic cancers, the heterogeneity of solid tumors warrants the application of TILs with a wide range of specificity. Recent advances in understanding TME, immune exhaustion, and immune checkpoints have paved the way for TIL therapy regimens. Nowadays, TIL therapy has regained attention as a safe personalized immunotherapy, and currently, several clinical trials are evaluating the efficacy of TIL therapy in patients who have failed conventional immunotherapies. Gaining favorable outcomes following TIL therapy of patients with metastatic melanoma, cervical cancer, ovarian cancer, and breast cancer has raised hope in patients with refractory solid tumors, too. Nevertheless, TIL therapy procedures face several challenges, such as high cost, timely expansion, and technical challenges in selecting and activating the cells. Herein, we reviewed the recent advances in the TIL therapy of solid tumors and discussed the challenges and perspectives.

## 1 Introduction

Tumor-infiltrating lymphocytes (TILs) are naturally-occurring mononuclear cells infiltrating the solid tumor microenvironment (TME), which might be referred all immune cells at the tumor site, too (1, 2). TILs history goes back to more than two centuries. In 1863, Virchow observed that neoplastic tissues contained leukocytes (3). In 1982, Steven Rosenberg, the father of adoptive cell therapy (ACT), isolated TILs from mouse models of tumors for the first time (4). He showed that combining cyclophosphamide, TILs, and interleukin (IL)-2 can improve 50-100% of colon adenocarcinoma-bearing mice with hepatic or pulmonary metastasis (5). This report has underpinned TIL therapy in treating advanced cancers. The first TIL therapy in humans was also conducted by his group in 1988, resulting in a 60% regression in metastatic melanoma (6).

Despite hematologic malignancies with lineage-specific markers, solid tumors are highly heterogeneous and do not possess an ideal tumor marker (7, 8). Targeting a tumor-associated antigen (TAA) leads to the predominance of tumor cells that do not express any special tumor marker (9). TILs are polyclonal cells with diverse receptors capable of detecting a wide range of TAAs, making them superior to genetically-modified immune cells in treating solid tumors. TILs can overcome tumors’ heterogeneity and immune escape and provide better clinical outcomes than chimeric antigen receptor (CAR)-T cells in treating solid tumors with high mutation rates, such as melanoma (10). TILs are mostly tumor-specific and can target even unknown tumor neoantigens within the TME, resolving the necessity of prior knowledge about TAAs or MHC restriction (11).

TILs are generally divided into intratumoral and stromal TILs (iTILs and sTILs). The iTILs are rare lymphocytes within tumor cell clusters, so their detection is complicated, while sTILs are frequently found in the tumor stroma and are easily detectable (2). Most TILs are effector memory T cells with high efficiency in proliferation and antitumor functions, are activated by TAAs *in vivo*, and can proliferate *in vitro* up to 10^5^ times (12). TILs are TME-infiltrated cells; therefore, they possess chemokine receptors necessary for migration toward the TME after injection (11). Another advantage of TILs to CAR-T cells is lower off-target toxicity, which probably returns to the negative selection of T cell receptors (TCRs) during T cell maturation (7).

Thus far, TIL therapy has shown significant clinical results in metastatic melanoma (13), cervical squamous cell carcinoma (CSCC) (14, 15), and cholangiocarcinoma (16), and its initial results in non-small cell lung cancer, colorectal cancer (CRC), and breast cancer (BC) have been promising (17–19). Besides, the prognostic role of TILs in multiple tumors has been confirmed and entered into clinical guidelines (20). Herein, we reviewed the latest prognostic and therapeutic advances of TILs in solid tumors and discussed the prospects of TIL therapy in cancer immunotherapy.

## 2 Comparison of TIL therapy with other adoptive T cell therapies

**Figure 1:**
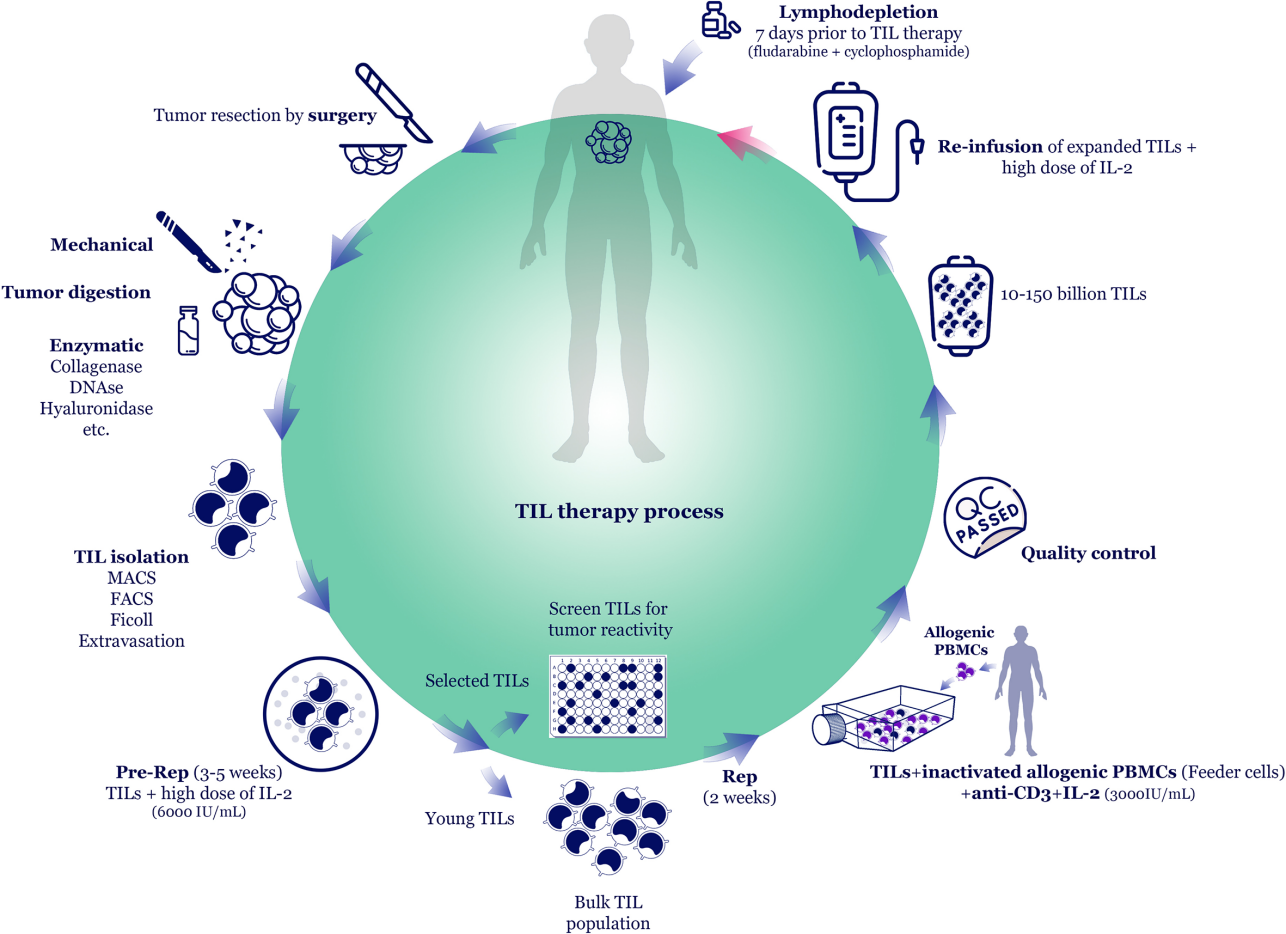
TIL therapy process. The most common TIL production method is TIL isolation from resected tumor tissue and expansion *in vitro* using rapid expansion protocol (REP). The tumor is excised by surgery and mechanically cut into small pieces. Then, tumor pieces can be further digested by adding enzymes, such as collagenases, DNAse, hyaluronidase, etc. TILs are isolated *via* Ficoll density gradient centrifugation or specific cell sorting methods, such as magnetic- or fluorescence-activated cell sorting (MACS/FACS). In the pre-REP phase, TILs undergo primary expansion in the presence of high-dose interleukin-2 (IL-2). In some methods, following pre-REP, the tumor-specific TILs are selected and further expanded (selected TIL method). However, to reduce *in vitro* culture period and to maintain TILs efficacy, some studies skip the TIL selection process and expand the bulk TILs (young TIL method). In REP, high dose IL-2, anti-CD3, and irradiated allogeneic peripheral blood mononuclear cells (PBMCs) as feeder cells are added to the TIL culture. The expanded cell products that passed the quality controls (sterility, negativity for blood-borne diseases, and phenotype checking) are ready to administer to the patient. Before TIL administration, patients undergo lymphodepletion, and 10-150 billion TILs are infused into patients along with high dose IL-2.

The advantages and disadvantages of different types of ACT and their brief protocols have been described in **Table 1**. TIL therapy depends on some procedures, including nonmyeloablative lymphodepletion and infusion of TILs, which are collected from a tumor mass and expanded *ex vivo* (**Figure 1**) (31). Although TILs separated from a resected solid tumor mass can recognize TAAs from their endogenous receptors, the inadequate number of obtained TILs is a limiting factor in cancer immunotherapy. *In vitro* administration of IL-2 as a T cell growth factor is a well-established protocol for expanding the isolated TILs (6). High-dose IL-2 exposure leads to a rapid proliferation of the lymphocytes, providing enough immune cells for ACT (13). TIL therapy is considered an effective therapeutic strategy in refractory metastatic melanoma, especially with the combination of nonmyeloablative lymphodepletion (13). TIL therapy relies on the infiltration of polyclonal T cells, capable of recognizing multiple TAA or unknown antigens. Recent studies on the TAA-specificity of TILs through peptide-loaded HLA multimers revealed low reactivity of TILs to the specific differentiation antigens.

**Table 1:** Clinical trials of TIL therapy.

Cancer type	Intervention	Phase	Sub	ORR(%)	Clinical trial identifier/Ref
**Metastatic melanoma**	TIL +IL2 + Non-myeloablative Lymphodepletion (NMA) chemotherapy + total-body irradiation (TBI)	II	93	72%	(21)
	TIL + NMA + IL2	Meta-analysis of 7 trials	332	43% ORR 15% CR	(13)
	TIL (LN-144) + IL2 + NMA + Pembrolizumab	II	NA	Recruiting	NCT03645928
	TIL (LN-145-S1) + IL2 + NMA	II	NA	Recruiting	NCT03645928
	TIL (IOV-4001) + IL2 + NMA	I/II	NA	Recruiting	NCT05361174
**Non-small cell lung cancer**	TIL (LN-145) + IL2 + NMA + Pembrolizumab/Ipilimumab/Nivolumab	II	28	21.4% 3.5% CR 17.8% PR	NCT03645928
	TIL (LN-145) + IL2 + NMA + Nivolumab	I	20	10% CR 60% PR with reduced tumor burden	NCT03215810 (17)
	TIL (LN-145) + IL2 + NMA	II	95	Recruiting	NCT04614103
	TIL (IOV-4001) + IL2 + NMA	I/II	NA	Recruiting	NCT05361174
**Ovarian cancer**	TIL + IL2+ cyclophosphamide	I/II	7	14.2% CR 57.1% PR	(22)
	TIL + IL2+ cisplatin	I/II	10	40% CR 50% PR	(22)
	TIL (MDA-TIL) + IL2 + NMA	II	3	No responder	NCT03610490 (23)
	TIL + NMA	III	17	82%	(22)
	Young TIL + IL2 + NMA + Pembrolizumab	II	NA	Recruiting	NCT01174121
	TIL (LN-145/LN-a45-S1) + IL2 + NMA + Ipilimumab/Nivolumab	II	NA	Recruiting	NCT03449108
	TIL + IL2 + NMA	I	6	Completed- No result yet	NCT02482090
**Head and neck squamous cell carcinoma**	TIL (LN-145) + IL2 + NMA + Pembrolizumab		NA	Recruiting	NCT03645928
**Breast cancer**	Neoantigen-specific TIL + NMA+ Pembrolizumab ≤ 4 doses	II	6	50% Tumor regression 16% CR (5.5 years) 33% PR	NCT01174121 (18)
	Young TIL + NMA + Pembrolizumab + IL-2	II	NA	Recruiting	NCT01174121
	TIL (LN-145) + NMA + IL-2	II	10	Recruiting	NCT04111510
	TIL + NMA	Early I	50	Recruiting	NCT05142475
	TIL (LN-145/LN-a45-S1) + IL2 + NMA + Ipilimumab/Nivolumab	II	NA	Recruiting	NCT03449108
**Advanced Colorectal cancer**	TIL+ 5-Fluorouracil-based chemotherapy	I/II	25	24-months survival rate= 55.6% vs 17.5% in controls	(19)
	TIL (MDA-TIL) + IL2 + NMA	II	8	No responder	NCT03610490 (23)
	TIL+ pembrolizumab	I	1	Terminated	NCT02757391 (24)
	Young TIL + IL2 + NMA + Pembrolizumab	II	NA	Recruiting	NCT01174121
**Pancreatic cancer**	TIL (MDA-TIL) + IL2 + NMA	II	5	20%	NCT03610490 (23)
	Young TIL + IL2 + NMA + Pembrolizumab	II	NA	Recruiting	NCT01174121
**Cervical carcinoma**	TIL+ Arm1: LN145 + IL2 Arm2: LN145 + pembrolizumab, IL2	II	27	44% ORR 4% CR	NCT03108495 (15)
	TIL + IL2 + Nivolumab		80	25% ORR 5% CR	(14)
**Cervical, vaginal,** **Anal carcinoma**	TIL+ HPV E6/E7 target + IL2	II	19	28%	NCT01585428 (25)
**Advanced RCC**	CD8+ TILs + IL2	III	77	9.9% ORR 1-year survival rate= 55% vs 47% in controls	(26)
	TIL + IL2 + NMA	I	6	Completed- No result yet	NCT02482090
	TIL + IL2 + NMA	I	4	25% ORR	(27)
	TIL + IL2	I	7	29%	(28)
	CD8+ TIL + IL2	I/II	55	34.6% ORR 9% CR	(29)
	CD8+ TIL + IL2	III	39	8% ORR	(26)
	TIL + IL2	III	6	30% ORR 30% CR	(30)
**Metastatic/Recurrent Advanced Solid Tumors**	TIL (GT201) + IL2 + NMA	I	30	Recruiting	NCT05430360
**Metastatic or unresectable epithelial tumors**	NEXTGEN-TIL + Non-myeloablative Lymphodepletion regimen + IL2	I	10	Recruiting	NCT05141474
**Endocrine Tumors**	Young TIL + IL2 + NMA + Pembrolizumab	II	NA	Recruiting	NCT01174121
**Osteosarcoma and other Bone and Soft Tissue Sarcomas**	TIL (LN-145/LN-a45-S1) + IL2 + NMA + Ipilimumab/Nivolumab	II	NA	Recruiting	NCT03449108
**Thyroid cancers**	TIL (LN-145/LN-a45-S1) + IL2 + NMA + Ipilimumab/Nivolumab	II	NA	Recruiting	NCT03449108

NA. Not available.

In contrast, the majority of TILs respond to unknown mutated epitopes that would not be a target for central tolerance during T cell differentiation, which is an advantage of TIL therapy compared to other ACT types (32). Following any upcoming therapeutic agent, toxicity is often a critical concern. TIL therapy has shown a favorable safety profile based on early-phase clinical trial studies. However, this form of ACT has significant side effects associated with co-administration protocols, including high-dose IL-2 and different types of chemotherapy regimens (13, 33). Toxicity manifestations might be observed immediately or take time to appear in some patients; however, the application of standard clinical practices can limit these side effects (34).

Altogether, the apparent advantages of TIL therapy are the stable and reproducible clinical outcomes for pre-treated patients with severe tumor progression who have been excluded from other ACT strategies (35). Nevertheless, TIL therapy encounters challenges; for example, it is the most individualized treatment, so a particular infusion product must be prepared for each patient. Good manufacturing practice (GMP) procedures and well-trained personnel are mandatory in this regard. In addition to the costly manufacturing procedure, sometimes the production procedure takes more than one month, which is undesirable for patients with rapid tumor progression (13).

In contrast to TIL therapy, tumor-specific T cell therapy mechanisms are based on developing genetically engineered T cells with accelerated antitumor activity. This is conducted by transferring genetic elements encoding a modified TCR or a synthetic CAR capable of recognizing specific tumor antigens. While different methods are available for developing genetically engineered T cells, the typical approach is based on collecting particular immune cells by leukapheresis before their genetic modification and eventual reinfusion. Similar to TIL therapy, a preconditioning regimen is often used prior to TCR-modified T cell therapy (31).

In TCR-modified T cell therapy, the specificity of T cells depends on modified TCR alpha and beta chains, leading to specific recognition of the tumor antigens (21). The generation of transduced T cells with TAA-specific TCR genes represents many advantages over TIL therapy. In this regard, TME-infiltrated T cells are not always available and would not expand to an adequate number necessary for cancer immunotherapy. On the other hand, the transduction of retrovirus-encoding modified TCR into the isolated peripheral blood lymphocytes can promote rapid access to produce massive TAA-specific T cells (32). Moreover, TCR-modified T cell therapy leads to a greater yield of activated neoantigen-specific T cells and has better proliferative potential than TIL therapy, which may display an exhausted phenotype of lymphocytes because of the frequent stimulation (36). The promising outcomes of the TCR-modified approach with differentiated melanoma/melanocyte antigen in patients with melanoma have been addressed in previous studies (37). Although TCR engineering is a desirable method for cancer immunotherapy, it has more limitations than TIL-based ACT. In this regard, recognizing only one specific tumor antigen allows the tumor cells to escape from TCR-modified T cells through the down-regulation of MHC class I or the tumor antigen, leading to antigen loss. In addition, autoimmune manifestation due to aberrant TCR recognition is a crucial concern. However, normal cells express these antigens at the lower level, but the high-affinity TCRs can represent significant binding to these restricted epitopes (38). Finally, unknown TCR specificity should be considered due to the modified TCR chains mispairing with endogenous TCR alpha and beta chains, leading to the high reactivity against self-antigens (38).

CAR-T cells combine antibody-based recognition with T cell functionality and cytotoxicity. The CAR-T cell structure depends on TCR signaling and the appropriate fragment of an antibody, targeting the molecules of interest expressed on the tumor cell surface. In contrast to TIL therapy and TCR modification strategy, CAR recognition is independent of peptide processing and antigen presentation on MHC molecules. Therefore, many cell surface antigens can be considered a potential CAR-triggering target which is the main advantage of CAR-T cell therapy over the other forms of ACT (39). Similar to transgenic TCR therapy, the source of T cells is not very important for CAR-T cell therapy, and T cells can be isolated from peripheral blood cells (40).

In contrast to the TIL therapy as a safe approach for the ACT, there are several safety risks associated with the CAR-T cell strategy, including *I.* on-target, off-tumor reactivity due to recognition of the same targeted antigen expressed on normal tissues, *II.* off-target reactivity due to the cross-reaction of CAR-T cells with non-specific peptides, and *III.* cytokine-release syndrome and immune effector cell-associated neurotoxicity syndrome, which is characterized by the significant secretion of inflammatory cytokines from non-specific immune cells (41).

## 3 TIL therapy procedure

The number and functionality of TILs are two critical factors determining the success of TIL therapy. The most common TIL production method is TIL isolation from resected tumor tissue and expansion *ex vivo* using rapid expansion protocol (REP) (**Figure 1**) (7). The tumor is excised by surgery and mechanically cut into small pieces. Then, tumor pieces can be further digested by adding enzymes, such as collagenases, DNAse, hyaluronidase, etc. TILs are isolated *via* Ficoll density gradient centrifugation or specific cell sorting methods, such as magnetic- or fluorescence-activated cell sorting (MACS/FACS). In this step, CD8^+^ T cells could be enriched, or Tregs could be depleted to enhance the antitumor effects of TIL therapy. Although mechanical and enzymatic digestions rapidly isolate TILs from the tumor tissue, they might damage TILs. Some studies suggest cutting tumor mass into 1 mm^3^ pieces and putting them into cell-culture media containing high-dose interleukin-2 (IL-2) for two weeks. It lets TILs gradually egress from tissue to the medium. However, this method requires a considerable amount of IL-2 because it should be replenished every 2-3 days.

In the pre-REP phase, TILs undergo primary expansion in the presence of IL-2. In some methods, following pre-REP, the tumor-specific TILs are selected and further expanded (selected TIL method) (11). However, to reduce *in vitro* culture period and to maintain TILs efficacy, some studies skip the TIL selection process and expand the bulk TILs (young TIL method) (42). In REP, high-dose IL-2, anti-CD3, and irradiated allogeneic peripheral blood mononuclear cells (PBMCs) as feeder cells are added to the TIL culture. Although TIL expansion occurs mainly *via* treatment with high doses of IL-2, various other *in vitro* expanding and stimulation methods such as cytokines (such as IL-15 and IL-21), costimulatory molecules, immune-checkpoint inhibitors (ICIs), as well as their co-culture with antigen-presenting cells (APCs) or feeder cells are available (1, 7).

Several methods have been used to detect and remove the residual tumor cells throughout TIL production following isolation from resected tumor tissue. In Rapid Expansion Protocol (REP), residual tumor cells die out after the culture of cells since the culture conditions only support lymphocytes (43). Moreover, obtained TILs by mechanical disaggregation and Ficoll-Hypaque density gradient centrifugation can be filtrated through nylon monofilament mesh to eliminate residual tumor cells (44). According to another study, residual tumor cells may be removed using mononuclear cells stimulated with IL-2 or cultivated in a serum-free environment (45). Besides, residual tumor cells can be removed from TIL products using FACS/MACS.

Generally, several techniques, such as allelic-specific oligonucleotide real-time quantitative polymerase chain reaction, immunohistochemistry, flow cytometry, and fluorescence *in situ* hybridization, can be employed to determine residual tumor cells (46, 47). Using immunohistochemical staining for S100, gp-100, and tumor markers melanoma-associated antigen recognized by T cells (MART-1), it is possible to detect the presence or absence of residual tumor cells (48). However, evaluating tumor markers by flow cytometry is more common in TIL manufacturing.

The expanded cell products that passed the quality controls (sterility, negativity for blood-borne diseases, and phenotype checking) are ready to administer to the patient. Before TIL administration, patients undergo lymphodepletion *via* chemotherapy and radiotherapy. Then, 10-150 billion TILs are intravenously infused into patients along with multiple high doses of IL-2. In most clinical trials, TIL therapy has been administered *via* the intravenous route, although few studies have reported other routes, such as intrapleural, intraperitoneal, intrathoracic, or intratumoral, based on the tumor location (49, 50).

## 4 TIL therapy for treatment of human solid tumors

### 4.1 Melanoma

Melanoma, the fifth leading cancer in the USA (51), develops from the malignant transformation of melanocytes, the pigment-producing cells found in the basal epidermis of the skin, the choroidal layer of the eye, inner ear, and leptomeninges (52). Invasive melanoma is one of the most lethal cancers and, despite comprising only 1% of skin cancers, accounts for over 80% of skin cancer deaths. Thanks to recent therapeutic approaches such as combinational ICIs with ipilimumab (anti-CTLA-4) and nivolumab (anti-PD-1), the overall melanoma mortality rate has declined, resulting in an improved 1-year relative survival rate for metastatic melanomas in most patients (53). Although FDA has not approved TIL therapy, it might be an appropriate regimen for ICI-resistant patients.

As mentioned earlier, the clinical development of TIL-based therapy began in 1988 on 20 patients with metastatic melanoma, leading to tumor regression in 40-60% of patients lasting 2-13 months (6). Then, the same group examined the efficacy of TILs in conjunction with high-dose IL-2, with or without cyclophosphamide, in 86 patients with metastatic melanoma (31). With an objective response rate (ORR) of 34%, no significant differences were seen between patients receiving or not receiving cyclophosphamide (35% versus 31%). The frequency of response to treatment has been associated with shorter culture duration, shorter doubling time, and higher lysis activity to autologous tumor targets.

In 2002, Dudley et al. showed that lymphodepletion with cyclophosphamide and fludarabine before TIL re-infusion led to *in vivo* clonal expansion of TILs and their prolonged response, as well as autoimmune melanocyte destruction in vitiligo and uveitis patients with IL-2 refractory metastatic melanoma. Six out of thirteen patients (47%) in this study had an objective partial response (54). After that, in three sequential clinical trials, they assessed the efficacy of TIL therapy in combination with IL-2 following a non-myeloablative lymphodepleting chemotherapy conducted by the administration of 60 mg/kg cyclophosphamide and 25 mg/m^2^ fludarabine with or without either 2 or 12 Gy of total-body irradiation, in 93 patients with measurable metastatic melanoma who followed-up for 62 months (55). Data indicated the superiority of total-body irradiation in conferring clinical benefits over chemotherapy alone, such that by adding 2 and 12 Gy of total-body irradiation, the ORR increased from 49% in the chemotherapy alone group to 52% and 72%, respectively. Twenty of the 93 patients (22%) had complete tumor regression lasting beyond three years, with 100% and 93% of 3- and 5-year survival rates, respectively. The high telomere length of the transferred TILs reflects their higher replicative capacity, which is strongly correlated with clinical response.

As previously mentioned, the higher telomere length of the infused TILs and higher expression levels of CD27 and CD28 are associated with survival and clinical efficacy (56, 57). Some studies have tried simplifying and shortening TIL preparation to generate young TILs with higher antitumor activity. In this regard, in a trial, administration of CD8^+^ enriched young TILs produced by a simplified method, omitting the personalized tumor-reactivity screening step, resulted in an ORR of 48%-58% in melanoma patients (58).

Besser et al. demonstrated that TIL therapy leads to persistent and complete responses in eighty patients with stage IV melanoma who were refractory to IL-2 or ipilimumab (59). Patients received unselected young TILs after a non-myeloablative lymphodepleting standard chemotherapy, followed by bolus high-dose IL-2. Thirty-two patients had been treated with ipilimumab before or after TIL transfer. The ORRs of 40% with five cytokine-release syndromes were seen among 57 evaluated patients. The 3-year survival rate was 78% in responding patients. Despite the lack of association between response to previous immunotherapy and the overall response to TIL therapy, the total count of transferred CD8^+^ cells, as well as the TIL culture duration independently predicted clinical outcomes.

More recently, Sarnaik et al. reported the safety and efficacy of lifileucel (LN-144), an autologous TIL product, in a phase II study sponsored by Iovance Biotherapeutics, Inc. in 66 patients with advanced melanoma who were refractory to prior treatment with ICI(s) and BRAF ± MEK inhibitors (60). After a non-myeloablative lymphodepletion regimen, patients were administered a single infusion of lifileucel followed by high-dose IL-2. The ORR was 36%, and the overall disease control rate was 80%. The ORR of 41% and the disease control rate of 81% were seen in the subgroup refractory to antibodies against programmed cell death protein 1 (PD-1) or PD-ligand 1 (PD-L1). In a multicenter phase III clinical trial on 186 patients with unresectable stage IIIC-IV melanoma who mainly were (86%) refractory to anti-PD1, the efficacy of 4 doses (3mg/kg) ipilimumab versus one dose (5×10^9^) TIL therapy was evaluated (61). The median PFS for ipilimumab was 3.1 months versus 7.2 months in TIL therapy group. The ORR was 21% and 49% in ipilimumab and TIL therapy groups, respectively. CR was 7% in the ipilimumab group, while in TIL group, it was 20%. Finally, the median OS was 18.9 months in ipilimumab versus 25.8 months in TIL group (61). Noteworthy, the grade ≥ 3 adverse effects were seen in 57% and 100% of ipilimumab and TIL groups, respectively. It suggests that TIL therapy might be a promising option for those unresponsive to ICIs (61).

### 4.2 Non-small cell lung cancer

Lung cancer, the first leading cause of cancer death globally, is divided into two major types, including small cell lung cancer (SCLC) and non-small cell lung cancer (NSCLC), which the latter accounts for ~90% of all cases (62). Although ICIs have revolutionized the treatment of NSCLC, evidence indicates that only a small proportion of patients experience objective responses to ICIs, and most of them show disease progression or grade 3–4 immune-related adverse events on immunotherapy (62). Several studies have demonstrated an association between higher TIL levels, improved recurrence-free survival, and reduced chance of systemic recurrence in NSCLC (17, 63). Also, NSCLC carries a high mutation load (64), leading to a high neoantigen level; furthermore, NSCLC is more likely to respond to ACT, including TIL therapy (17).

The first cancer TIL therapy trial was reported by Kradin et al. in 1987 on NSCLC patients (65). Although five of seven patients experienced cancer reduction, none of them achieved an objective response. The next trial by Kradin et al. on eight NSCLC patients did not result in measurable responses (28). In 2018, Ben-Avi et al. assessed the feasibility of TIL generation according to the well-established melanoma TIL protocol in five patients with advanced-stage NSCLC undergoing thoracic surgery. Despite the small size of the tumors, they reported a successful TIL establishment in all of them (63).

More recently, in a phase I study, Creelan et al. evaluated the safety and efficacy of autologous TILs in combination with nivolumab in twenty patients with advanced NSCLC following disease progression on nivolumab monotherapy (17). Patients received a single TIL infusion preceded by standard lymphodepleting chemotherapy, followed by IL-2, and then nivolumab maintenance. Eleven out of thirteen patients showed tumor regressions with a median best change of 35%, and three had confirmed responses, including two complete responses lasting for 1.5 years. However, none of the patients achieved median OS. Despite these controversial results from very few small studies on NSCLC patients, TIL therapy might still be a promising candidate for the management of lung cancer. It will be clarified upon releasing the results of several ongoing clinical trials exploring the clinical efficacy of TIL therapy alone or in combination with ICIs in lung cancer (NCT04614103, NCT03215810, NCT03903887, NCT04919616, NCT03645928, NCT00019084, NCT03407040, and NCT04677361).

### 4.3 Ovarian cancer

Ovarian cancer is the eighth major cause of cancer-related mortality in women globally. Among different histological subtypes, epithelial ovarian cancer (EOC) accounts for ~90% of ovarian cancers (66). Despite significant advances in therapeutic strategies, the prognosis for EOC remains poor, and disease recurrence occurs in a considerable portion of patients within 2–3 years (67). Indeed, despite the great hope placed on ICIs in cancer therapy, their use in most EOC patients has not yielded clinically meaningful results so far (68). The association of the iTILs with good clinical outcomes has triggered TIL therapy ideas in EOC; nevertheless, the available trial results have not shown any significant clinical efficacy. In 1991, Aoki et al. published the first results of TIL therapy with or without chemotherapy in 17 patients with advanced or recurrent EOC (22). In TIL only group (7 patients), the ORR was 75%, and one patient had a complete response. In TIL plus chemotherapy (10 patients), 90% ORR and seven complete responses were obtained. Two trials on intraperitoneal TIL therapy by Freedman et al. did not result in detectable responses, except for some reduction of CA125 level in ascites and blood of a few patients (69). In a phase I trial (70), Fujita et al. compared the clinical efficacy of TIL therapy in the first-line setting in EOC with standard first-line treatment. Thirteen patients received TIL infusion following primary debulking surgery, then platinum-based adjuvant therapy. As a control group, eleven patients were treated with only standard first-line treatment. The 3-year disease-free survival (DFS) rates of 82.1% against 54.5% were seen in the TIL group and controls, respectively.

In a pilot study at National Center for Cancer Immune Therapy (71), Pedersen et al. treated six platinum-resistant patients with a single TIL infusion after standard lymphodepleting chemotherapy followed by high doses of IL-2. Clinical responses were limited and primarily short-lived, and infused TILs expressed exhaustion markers, including lymphocyte-activation gene-3 (LAG3) and PD-1. More recently, the same group assessed the combination of TIL therapy with ICI sequentially (72). Six patients with late-stage EOC received an infusion of TILs preceded by ipilimumab followed by low-dose IL-2 and nivolumab. Partial response was seen in one patient, which prolonged for 12 months; the other five patients experienced short-lived stable disease. Similar to the previous study, 90-100% of infused TILs expressed LAG3. The engagement of LAG3 on T cells with MHC-II on cancer cells usually results in limited clinical outcomes. Hence, they conducted a phase I/II study on 18 patients with advanced ovarian cancer, in which they added relatlimab (anti-LAG3 antibody) to the TIL therapy regimen (72) to unleash T cell antitumor activity by inhibiting the LAG3-MHC-II interaction (NCT04611126). Preliminary results of phase I/II ongoing clinical trial testing the feasibility and safety of TIL therapy during carboplatin-paclitaxel chemotherapy with or without interferon (IFN)α in 12 patients with recurrent platinum-sensitive EOC showed that ten patients (83%) achieved ORR of 83% and two experienced stable disease (73).

There are several ongoing clinical trials of TIL therapy alone or in combination with other therapeutic strategies in patients with EOC (NCT03412526, NCT03610490, NCT03318900).

In conclusion, despite feasibility and tolerability, TIL therapy in EOC had limited success. Some possible explanations for this low clinical efficacy could be inefficient *ex vivo* expansion, expression of exhaustion markers, such as PD-1, LAG3, and suboptimal lymphodepleting chemotherapy, or IL-2 support (71, 72).

### 4.4 Head and neck squamous cell carcinoma

Head and neck squamous cell carcinoma (HNSCC) is a heterogenic group of cancers developing from the mucosal epithelium in the oral cavity, pharynx, and larynx (74). According to causative factors, HNSCC is classified into two categories: human papillomavirus (HPV)-positive and HPV-negative cancers. Also, the Epstein-Barr virus (EBV) has been associated with a subtype of HNSCC, so-called nasopharyngeal cancer (NPC) (74). Since viral oncoproteins are expressed in HPV and EBV-associated cancers, they are ideal targets for ACT (74).

Despite the great hope placed on ICIs in HNSCC, its response rates remain less than 20% (75). Increasing evidence indicates the feasibility of patient selection in HNSCC for TIL therapy and gives the green light to its clinical testing, confirming a higher TIL number as a significant prognostic factor in the OS of both HPV-positive and HPV-negative patients (76). In addition, HPV-associated oropharyngeal cancers harboring viral oncoproteins such as E6 and E7 as a target for TIL therapy (77). Recently, in a clinical trial study (phase II) by the National Cancer Institute, the clinical efficacy of TIL therapy was evaluated in metastatic HPV-related cancers (25). Patients received a single TIL infusion, preceded by standard lymphodepleting chemotherapy, followed by high-dose IL-2. The ORRs of 28% and 18% have been observed in cervical cancer and non-cervical HPV-related cancer groups, respectively; one of whom has been a patient with HNSCC with lung metastases who experienced a response lasting five months (25).

More recently, in a clinical phase I/II trial, Kverneland et al. evaluated the efficacy of TIL therapy supported by ICIs in 25 patients with different progressive metastatic cancers, one of whom was an HPV-positive HNSCC patient (51). Patients received a single TIL infusion preceded by ipilimumab and nivolumab, as well as chemotherapy, followed by nivolumab and low-dose IL-2. They reported sizeable tumor regressions of 30%-63% in 5 patients, including confirmed partial response (16%) in two patients with HNSCC (51).

Based on these results, a phase II multicenter clinical trial is ongoing to evaluate the efficacy of a single autologous TIL infusion (LN-145/LN-145-S1) followed by IL-2 after a standard lymphodepleting regimen in patients with recurrent and metastatic HNSCC (NCT03083873). Another phase II trial is ongoing to explore the efficacy of combination TIL therapy with pembrolizumab in PD-1-naïve patients with advanced, recurrent, or metastatic HNSCC (NCT03645928), and preliminary results from 12 patients have shown an ORR of 42.9% (78).

Also, there is an ongoing phase I clinical trial of tumor growth factor (TGF)-β resistant, EBV-specific T cells for treating EBV-positive NPC (NCT02065362). The obtained results motivated researchers to evaluate the clinical efficacy of TIL-based ACT in the treatment of NPC. In a phase I study, Jiang et al. assessed TIL therapy’s safety and antitumor activity following concurrent chemoradiotherapy in patients with EBV-induced locoregionally advanced NPC and reported sustained antitumor activity and anti-EBV immune responses following TIL therapy (79). Twenty patients received a single dose of TIL infusion following concurrent chemoradiotherapy. Nineteen patients experienced an ORR and DFS longer than 12 months after TIL infusion (79). The results of these clinical trials will shed light on the potential TIL therapy in HNSCC and might change the landscape of its management, especially in HPV and EBV-positive subgroups.

### 4.5 Breast cancer

The first evidence of TILs and their association with better clinical outcomes in BC was reported in 1992 (80). TILs frequency is approximately 10% in luminal BCs, 15% in HER2^+^ BCs, and 20% in TNBC (2). Moreover, in 20-28% of TNBCs, called lymphocyte-predominant BC (LPBC), TILs constitute >50-60% of the tumor stroma (2). Broadly speaking, lymphocyte infiltration is associated with a better prognosis in all BC types, especially in TNBC and HER2^+^ BC (81). Studies show that for every 10% increase in breast TILs, there is a 15-20% reduction in recurrence and mortality rate (2). Breast TILs approximately comprise of 70-80% T cells (2/3 are CD4^+^ and 1/3 are CD8+), 20% B cells, <5% macrophages, <5% NK cells, and 1% DCs (2). Tissue-resident memory T cells (TRMs) are CD8^+^ CD69^+^ CD103^+^ cells with a greater cytotoxic potential than effector CD8+ cells (82). TRMs comprise ≈40% of CD8^+^ TILs in BCs. TRMs <20% of the CD8^+^ T cells are associated with poor prognosis, while increasing TRMs to >60% of the CD8^+^ T population improves the recurrence-free survival (RFS), OS, and treatment responses in TNBC (82). Hence, TRMs have the potential as a predictive marker and a therapeutic target in TNBC (83).

Regulatory T cells (Tregs) are principal immunosuppressive cells in the TME (1). They infiltrated tumors following the PITPNM3 receptor response to CCL18 secreted by tumor-associated macrophages (TAMs) (84). Increased CCL18 levels, along with increased Tregs and follicular regulatory T cells, are strongly associated with increased relapse risk and lower RFS and OS (85, 86). Regarding the fact that the Tregs depend on IL-2, the use of IL-2 superkine (fusion of IL-2 and Fc of IgG2) and PEGylated IL-2 can decrease Tregs and stimulate CD8^+^ T cells and NK cells (87, 88). *In vivo* anti-CD25 can also deplete CD25^+^ Tregs for a long time (89). We have also reported that using pentoxifylline (a methyl xanthine derivative) can reduce the Treg proportion and enhance antitumor responses in an IL-2-mediated expansion of TILs (1). Interestingly, chemotherapy regimens have a more destructive effect on Tregs than CD8^+^ T cells (90). In contrast, Tregs are radio-resistant and prevail after radiotherapy (91). Cytotoxic T-lymphocyte-associated protein 4 (CTLA4) is a major inhibitory molecule of Tregs. Administration of anti-CTLA4 as monotherapy or combined with anti-PD1 resulted in ORR=12% and 12-month OS in 36% of chemotherapy-resistant patients (92).

Tumor-infiltrated CD57- NK cells, along with the high expression of CD155, can predict the complete pathological response (pCR) after treatment and improve OS in all BC patients (93, 94). Generally, BCs that respond better to trastuzumab (anti-HER2 antibody) have more NK cells and potent antibody-dependent cellular cytotoxicity (ADCC) (95). Activation of NK cells *in vivo* or *ex vivo* with IL-2, IL-15, and IL-12, as well as using CAR-NK cells, are NK-based therapeutic methods in BC (96, 97). The safety and efficacy of a CAR-NK cell produced by binding trastuzumab to NK cells are currently under investigation in HER2^+^ patients (NCT04319757) (98).

B cells are highly infiltrated in 20% of BCs, accounting for about 40% of total TILs (2). Tumor-infiltrating B cells (TIBs) can undergo affinity maturation at the tumor site to secrete high-affinity apoptosis-inducing IgG against tumor antigens (99). B cells also act as APC to stimulate T cells. However, B cells affected by CD40, Toll-like receptor (TLR) ligands, and inflammatory cytokines may become regulatory B cells (Bregs) (100), which are able to suppress immune responses and induce Treg differentiation (100). PD-L1^+^ TIBs were significantly associated with improved survival and pCR after treatment (101). Contrarily, the increase in CD19^+^ CD24^hi^ CD38^hi^ Bregs in BC is associated with higher Tregs and lower Progression-free survival (PFS) (102). Using CXCR5-targeted CpG ODN (103), signal transducer and activator of transcription 3 (STAT3)-inactivating resveratrol (104), and IL-10 depletion (100) can reduce Bregs and Tregs. More preclinical and clinical studies are needed to determine the prognostic and therapeutic potentials of TIBs.

Various methods are being studied to improve the amount, composition, and function of TILs. Radiotherapy and chemotherapy improve the infiltration and function of TILs by inducing immunological death (105). Cancer vaccines are also a promising way to strengthen TILs that have been shown to improve the three-year PFS from 31% to 76.9% in phase II/IIIA clinical trial on progesterone receptor (PR-)/estrogen receptor (ER-) patients (106). PD-L1 is expressed in about 60% of BCs and is positively associated with higher TIL levels (107). PD-L1 expression level could be >60% in TNBCs, highlighting the success of ICIs in TNBC (108). Accordingly, anti-PD-L1 with ORR=28% showed better results than anti-PD-1 with ORR=16% and anti-CTLA4 with no significant response (109). Atezolizumab (anti-PD-L1) and pembrolizumab (anti-PD-1) are currently the only FDA-approved ICIs for treating TNBC (110, 111). In the field of CAR-T cells, the use of MUC1-specific CAR-T cells (a protein upregulated in 95% of BCs) exhibited promising results and is currently in phase I clinical trials for treating metastatic BCs (NCT04020575) (112). It has been observed that increased RAS/MAPK signaling is associated with decreased lymphocyte infiltration in TNBC. Accordingly, MEK inhibitors increased lymphocyte infiltration, MHC-I and MHC-II, and PD-L1 expression in the TME (86). This finding suggests that combining MEK inhibitors with ICIs could be an interesting option for synergistic antitumor effects. Additionally, targeting other ICs such as T cell immunoglobulin and mucin-domain containing 3 (TIM3), indoleamine 2,3-dioxygenase (IDO), and the adenosinergic pathway has shown promising results in improving the function of TILs in TNBC, which should be the subject of future investigations (113–115).

### 4.6 Colorectal cancer

CRC is the third most common cancer worldwide. The type and density of TILs are important histopathological features of CRC and strongly affect the tumor progression and OS rate (116). Several studies showed that high levels of TILs are associated with a better prognosis in CRC. On the other hand, reduced TIL infiltration is associated with metastasis and the spreading of the tumor cells into the blood, lymphatic vessels, and the perineural space (117, 118).

TILs in CRC are a mixture of T cells, B cells, NK cells, macrophages, and other immune cells that impact the prognosis of CRC. Thus, their population can serve as a prognostic biomarker, and CRC could be an attractive target for immunotherapy (119).

High frequency of T cells (CD4^+^ or CD8^+^) in CRC tissue TILs is associated with a lower risk of metastasis, significantly improved prognosis, reduced relapse rate, and longer DFS and OS (120). CD8^+^cytotoxic T cells in the tumor epithelium can destroy tumor cells by recognizing the tumor antigens and directly suppressing metastasis. The existing studies showed a correlation between the prognosis of CRC and the number of CD8^+^ T cells. Therefore, a lower CD8^+^ cell number is associated with lower DFS and relapse rates (121). CD8^+^ T cell frequency predicts an effective response to chemotherapy, and also, there is a positive correlation between high pretreatment CD8^+^ T cell density and response to neoadjuvant chemoradiotherapy (122). In patients with high microsatellite instability CRCs, the high numbers of CD8^+^ TILs due to their specific neoantigen load were accompanied by an improved response to anti-PD-1 antibodies (123).

Effector memory T cells are responsible for long-lasting protection against tumors and are defined by the presence of CD3, CD8, CCR7, CD45RO, CD27, and CD28 markers. Infiltration of memory T cells is related to the absence of metastatic invasion and improved clinical outcomes (124). Intriguingly, the accumulation of Tregs in the CRC is associated with a favorable prognosis, while a higher ratio of CD8^+^ cells to FOXP3 seems to improve the prognosis (125).

The presence of TIBs is accompanied by infiltration of CD8^+^ T lymphocytes and has a positive prognostic role in CRC (126). CD20^+^ TIBs play different roles in antitumor immune response, such as the production of anti-TAA antibodies, cooperation with cytotoxic T lymphocytes by producing cytokines that can support T cell responses, and antigen-presentation to T cells to induce a cellular immune response (127). The favorable prognostic value of CD20^+^ TIBs in CRC synergizes with the prognostic effects of CD8^+^ T lymphocytes (128). Contrastingly, there is a negative correlation between tumor-associated neutrophils (TANs) and TAMs density and CRC patient prognosis (129).

These findings suggest that an active immune response correlates with favorable survival and provides a rationale for TIL therapy in CRC. Previous reports demonstrated the possibility of isolating and expanding sufficient numbers of TILs from CRC patients, which provides a rationale for advancing personalized immunotherapy in CRC (130). In a clinical trial (NCT01373047) of 16 patients with CRC, infusion of expanded sentinel lymph node (SLN)-derived CD4^+^ T helper 1 (Th1) cells induced an antitumor response, and complete tumor regression occurred in four of nine stage IV patients with distant metastases (131).

In another clinical trial conducted by Gardini et al. in the 1990s, 14 CRC patients with positive carcinoembryonic antigen (CEA) and liver metastases were treated with IL-2-expanded TILs. Findings showed no significant difference in DFS between the conventional chemotherapy and TILs group (132). A phase I/II study of SLN T cell-based therapy in patients with stage IV CRC showed that the survival rate of patients who received SLN T cell transfusion was significantly higher than the controls (19).

Recently, the successful application of TILs in CRC patients was reported in 2016 by Rosenberg’s team. In this study, KRAS G12D-specific CD8^+^ T cells were expanded from metastatic lung lesions of a CRC patient, and following TIL therapy, results showed eradication of 6 of 7 lung metastases. One tumor-progressing patient expressed the mutated KRAS G12D and did not genetically encode the HLA-C*08:02 allele. Harvesting sufficient TILs from CRC samples is challenging because relatively few effector cells infiltrate CRC tumors (133). Currently, CD8^+^ T cell therapy with pembrolizumab is an active clinical trial to treat CRC.

### 4.7 Liver cancer

The most common type of liver cancer (90%) is hepatocellular carcinoma (HCC). TILs play a major role in the prognosis and immunotherapy of HCC. Some TIL subsets show significant prognostic values on treatment and survival outcomes so that they can serve as prognostic biomarkers. Foxp3^+^, CD8^+^, and CD4^+^ T cells are the most widely analyzed subgroups of TILs in HCC (134). The high frequency of TILs in the invasive margin, intratumoral, and perivascular areas is associated with improved OS and RFS in HCC patients. In addition, TILs within the tumor or perivascular area are positively associated with the outcome of HCC patients, and deleting the exhausted effector T cells expressing PD-1 reduces the incidence of HCC so that the density of CD8^+^ cells can be a prognostic marker and can predict the treatment outcome in HCC (135).

Increased density of CD4^+^ TILs is also associated with better outcomes and acts as a protective factor in HCC. Disruption of CD4^+^ cells impairs CTL activation and correlates with poor prognosis and high recurrence of HCC (136). Contrarily, the frequency of Foxp3^+^ Tregs in peripheral blood and tumor tissue is associated with poor prognosis and invasiveness of HCC (137). The decreased frequency of PD-1^+^ Foxp3^+^ Tregs after administration of sorafenib, a multikinase inhibitor, enhances the antitumor immune responses. In addition, ICIs targeting CCR4, PD-1, LAG3, TIM3, and glucocorticoid-induced TNFR-related protein (GITR) on the Treg surface enhance the TILs’ antitumor function in HCC (138).

B lymphocyte subtypes, including naive B cells, CD20^+^ B cells, CD27^-^ isotype-switched memory B cells, IgM^+^ memory B cells, and plasma cells, are present in HCC, defined as TIBs, all of which show reduced count and functionality compared to normal tissue (139). A reduced number of naive B cells and CD27^-^ isotype-switched memory B cells are predictive factors for the progression of HCC. TIBs secrete IL-12 and IFN-γ and increase the infiltration of CD8^+^ T cells and NK cells to eliminate tumor cells (140).

The density of NK cells and macrophages is also associated with the prognosis of HCC. A low intratumoral CD56^+^ NK cell subset correlates with shorter DFS and OS outcomes (141). Peritumor-activated hepatic stellate cells predict poor clinical outcomes in HCC after therapeutic resection (142).

HCC patients with prominent lymphocytic infiltration who were surgically resected had a 38.6% lower recurrence rate and a 34.9% higher five-year OS than patients with weak lymphocytic infiltration. Considering the significant correlation between TILs and HCC prognosis, using TILs expanded from HCC can be a promising treatment (143). The feasibility of TIL therapy was demonstrated in phase I clinical trials in HCC patients. In a randomized clinical trial, TIL infusion improved RFS after liver resection in 150 HCC patients (144). Jiang et al. conducted the only phase I clinical trial of TIL therapy as a new treatment strategy for HCC patients. In this study, 15 HCC patients received autologous TILs after tumor resection. After a median follow-up of 14 months, 15 patients (100%) were alive, 12 patients (80%) showed no evidence of disease, and three patients had tumor recurrence. Despite this report of relatively high antitumor efficacy and low toxicity of TILs, there are no other clinical studies involving TIL therapy in HCC (145).

### 4.8 Other solid tumors

The critical application of TIL therapy was also reported in other types of solid tumors, including gastric carcinomas, gynecologic, and urological cancers. High infiltration of CXCR3^+^ immune cells in EBV-associated gastric carcinomas is associated with improved prognosis and is considered a discrete prognostic factor for RFS but not OS (146, 147). In contrast, the expression of CCR7 and the presence of PD-L1^+^ exhausted T cells are associated with a poor prognosis of gastric carcinomas (148).

Few studies report the presence of tertiary lymphoid structures in pancreatic ductal adenocarcinoma (PDAC) and their correlation with OS and PFS (149). In these patients, the immunosuppressive features of Th2 cells contribute to the rapid progression of tumors and reduction in OS (150). Some immunohistochemical studies reported that high recruitment of TAMs, TANs, and FOXP3^+^ Tregs was associated with a worse prognosis in PDAC patients (151, 152). However, similar to gastric carcinoma, infiltration of CD8^+^ T cells and CD20^+^ B cells is correlated with improved PFS in PDAC (152, 153).

There is not enough knowledge about the prognostic value of TILs in gynecologic cancers. Intra-epithelial CD8^+^ T cells and stromal CD3^+^ T cells may have prognostic significance (154). Workel and colleagues reported that intra-epithelial PD-1^+^ CD8^+^ T cells were associated with a favorable prognosis in vulnerable patients to endometrial carcinoma (155). A comprehensive systematic review of the ACT in gynecologic cancers has reported 41.4% ORR, 57.6% of disease control rate, 31.4% of disease stability rate, and 46.0% toxicity rate for TIL therapy in 238 patients with grade III/IV gynecologic cancers (156).

The prognostic significance of TILs in three urological tumors, including renal cell carcinoma (RCC), prostate cancer, and urothelial bladder carcinoma (the most common type of bladder cancer), is still unclear. Early reports in RCC indicated that TILs mainly included functional CD4^+^ T cells, effector memory CD8^+^ T cells, and NK cells with a limited population of B cells (157). Several studies have shown that increased T cell numbers are associated with tumor recurrence and a worse prognosis of RCC (158, 159). Moreover, Tregs’ presence can dampen T cells’ antitumor function (160). The studies documented before 2009 about TIL therapy in RCC seems disappointing because of the lack of reactivity of TILs against RCC (161). However, in recent years, several published studies demonstrated that manipulation of TILs can result in the generation of TILs with high tumor reactivity in recognizing or killing autologous tumors (162–164). Nevertheless, additional clinical studies are warranted.

The constituent of TILs in prostate cancer is still questioned and sometimes conflicting. Usually, TILs are scarce in the prostate TME. Although several studies have reported that CD8^+^ T cells are the predominant TIL population in prostate cancer, some studies reported a high proportion of CD4^+^ T cells and Tregs in prostate cancer (165, 166). Despite reports showing that high TIL infiltration is associated with recurrence, metastasis, and poor OS (167, 168) in prostate cancer, Yang et al. reported an improved 5-year OS in patients with a high number of TILs compared to patients with low TILs (169). The prognostic value of TILs on urothelial bladder cancer depends on the status of the disease and the region where they were measured (170). The increase in T cell frequency is correlated with worse prognosis and poor OS in non-muscle-invasive bladder cancer (171).

In contrast, a high proportion of T cells in muscle-invasive bladder cancer is associated with better clinical outcomes (172). Moreover, Wahlin et al. reported that CD8^+^ T cells and Tregs are associated with improved outcomes (173). The inconsistent findings of such studies indicate the necessity of further investigations.

## 5 Challenges & limitations of TIL therapy

**Figure 2:**
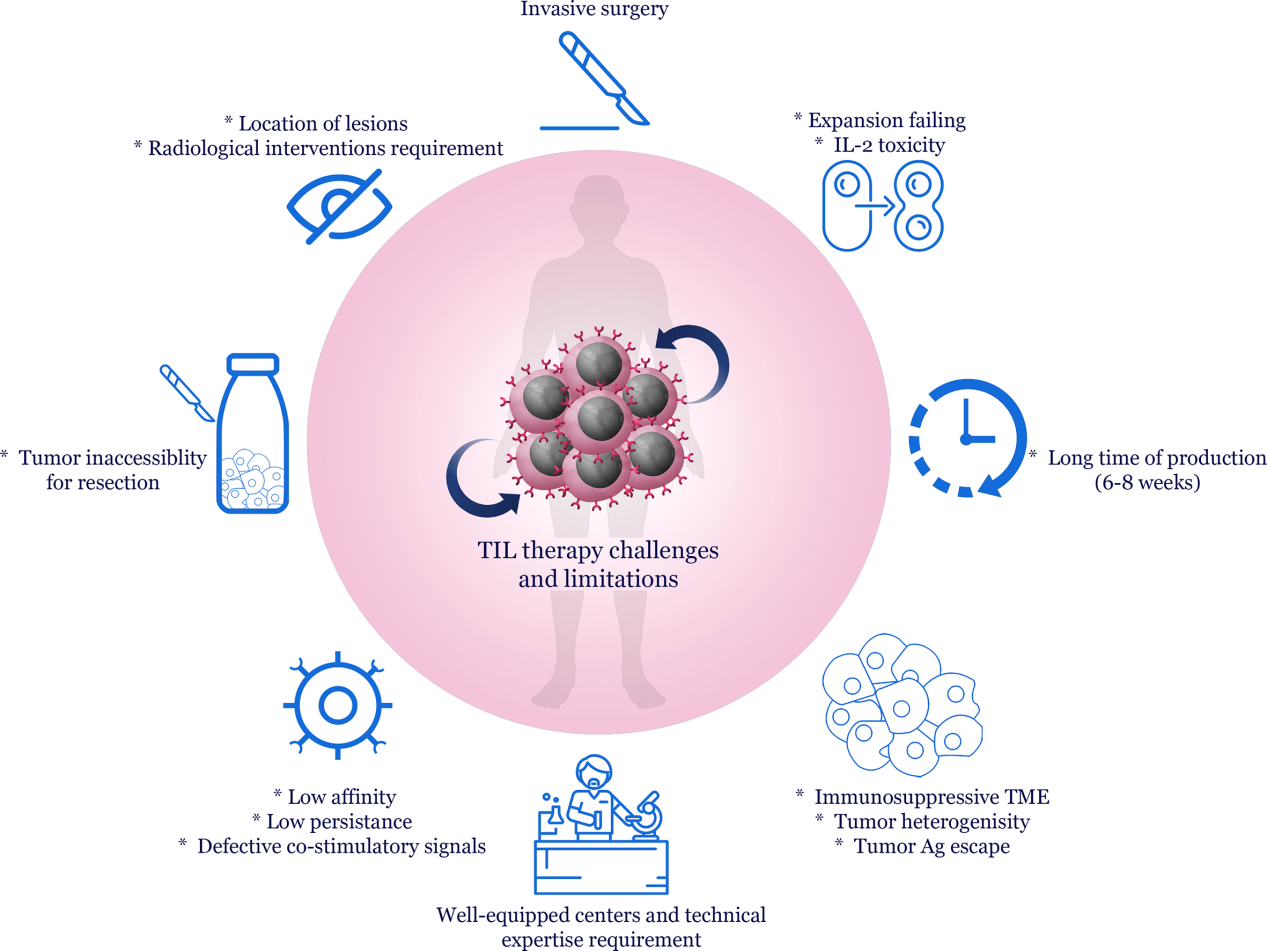
Challenges and limitations of TIL therapy. Immunosuppressive TME, tumor heterogeneity and immune system escape mechanisms are considered the main challenges to immunotherapy and cell therapy of human malignancies. Resection of the tumor mass may be difficult due to the inaccessibility of the tumor tissue. Surgery is considered an invasive method for the patient. Moreover, expanding TILs requires appropriate equipment and technical experience after separating them from the tumor tissue. Despite these cases, *in vitro* expanding TILs sometimes fail, and high doses of IL-2 for expansion may also be associated with toxicity. On the other hand, the preparation time of TILs is about 6 to 8 weeks, which affects the effectiveness of treatment in highly progressive tumors. Additionally, the low affinity of TILs isolated from tumor tissue, low persistence, and defects in co-stimulator molecules are other problems with TIL therapy.

ACT faces obstacles, including immunosuppressive TME, tumor heterogeneity, antigen escape, and ineffective trafficking to the tumor site; nonetheless, TILs have overcome some of these challenges because they are isolated from the tumor site (174). Despite TIL therapy’s benefits in treating solid cancers, this therapeutic method is associated with challenges and limitations (175) (**Figure 2**). The first step in TIL therapy is isolating TILs, which requires surgery to resect tumor tissue, and this invasive method can be distressing and even risky for patients with cancer (21). Furthermore, the tumor is not always accessible for resection, such as a subcutaneous nodule or deposit (176). Determining the exact location of lesions for tumor resection sometimes requires radiological interventions, the equipment for which is not available everywhere. On the other hand, following tumor resection, only a part of the TILs can be isolated and expanded. In the case of melanoma, about one-third of the isolated TILs fail to expand (177). In addition, preparing an exclusive TIL culture with antitumor activity for each patient and the necessity to access special and well-equipped centers, as well as technical expertise for TIL culture and expansion, are other obstacles in TIL therapy (58, 178). Another limitation of TIL therapy is the 6–8-week period for expanding and preparing these cells, which regarding the rapid progress of the tumor in the patient’s body, is considered a long time.

For this reason, many patients miss the optimal treatment window, or their treatment is not completed (55). Therefore, designing protocols to reduce TILs production time is also imperative. In this regard, “CD8^+^ enriched young TILs” were employed to overcome this problem and give a comparable response rate to conventional TIL therapy. In this method, TILs are briefly grown and reinfused into the patient, ignoring the personalized tumor-reactivity assessment phase, which requires the co-culture of TILs with their autologous tumor cells. Furthermore, these tumor cells must be freshly cryopreserved following tumor resection (58). Additionally, genetic engineering approaches can help to produce tumor-reactive T cells and compensate for the lack of TIL availability in solid tumors (174).

Rapid expansion protocol, which uses IL-2, OKT-3, and irradiated feeder cells isolated from the autologous patient or multiple donors, is another way of expanding TILs (**Figure 1**). Nonetheless, this method can achieve TILs with different potency due to variations in the expression of co-stimulatory molecules on the surface of personalized T cells in different donors (178). Genetically-enhanced artificial APCs were also fabricated to develop a standardized “off-the-shelf” platform for the fast expansion of tumor-reactive TILs from patients with limited TIL numbers (179). Evidence revealed that the low persistence and reduced function of reinfused TILs are also considered other factors of the failure of TIL therapy because most of the TCRs isolated from patients with cancer have a relatively low affinity for tumor antigens (37). In this context, researchers have used high-avidity engineered T cells to enhance antitumor responses, which can be associated with undesirable adverse effects (180). Therefore, finding a way to enhance the function of low-affinity T cells without TCR genetic manipulation is still a necessity.

Co-stimulatory signals are often defective in TILs, and their persistence and degranulation capability are affected in the TME (181). Using co-stimulators could be beneficial for strengthening and sustaining the cytotoxic effects of TILs (182). However, studies show that co-stimulation is challenging because not all effector T cells express CD28 and will be regarded as CD28^-^ TIL subsets (183). On the other hand, tumor cells also reduce the expression of CD80 and CD86 and increase the expression of inhibitory molecules such as PDL-1, which neutralizes TIL-dependent antitumor responses (184, 185). The importance of using molecules such as tumor necrosis factor receptor superfamily-9 (TNFRSF-9, 4-1-BB) as a co-stimulator in CAR-T cells is determined here (186). A look at the protocols of TIL therapy showed that administrating IL-2 to expand TILs is a common regimen. However, administering high doses of this cytokine in TIL therapy is associated with several side effects. In this context, studies have claimed that with low doses of IL-2 or other cytokines, patients under TIL therapy experienced fewer side effects and preserved effective partial or complete responses (13, 33). Another challenge of TIL therapy is the speed of the emergence of novel immunotherapeutic methods, such as ICIs, which can lead to the reprogramming of the TME by inhibiting immunosuppressive signals. As a result, infiltrated T cells can have constant and improved antitumor activity, along with less exhaustion (187).

Collectively, it can be argued that despite the advantages of TIL therapy, finding solutions to optimize protocols related to the expansion of tumor-reactive TILs and reduced production time can improve the capabilities of this therapeutic method. Correspondingly, combining therapies can undoubtedly increase cancer therapy’s effectiveness in a synergistic fashion.

## 6 Combination of TILs with other anti-cancer therapies

Despite promising clinical findings following TIL therapy in solid tumors, many patients do not respond to the treatment (188). Therefore, researchers are looking for alternative therapeutic options using combination therapies, the most important of which are mentioned in this section (**Table 2**).

**Table 2:** The most important combination therapies with TILs.

Combination strategy With TILs	Structure	Function	Type of tumor	Ref
**Chemotherapy**	Cisplatin	Increased the CR rate from 30% to 70% with RFS of 15 months	Ovarian	(22)
	Methotrexate + Cisplatin + Doxorubicin	Significantly increased DFS and OS compared to monotherapy with no additional adverse effect	Osteosarcoma	(189)
**Immune Checkpoint Inhibitors** **(ICIs)**	Anti-PD1-TIL therapy	Improved prognosis and enhanced survival time	Cervical	(14)
	Anti-PD1-TIL therapy	Reduced adverse effects and enhanced safety	metastatic osteosarcoma	(190)
	Anti- CTLA4-TIL therapy	Improved antitumor immune response and increased survival time	Metastatic Melanoma	(191)
	Anti PD1- & Anti-CTLA4- TIL therapy	Manageable cytotoxicity and sizable tumor regression	Heavy and neck cholangiocarcinoma	(72)
**Oncolytic Virus** **(OV)**	Adenoma Virus-TIL therapy	Desired TILs delivery system and increasing its cytotoxicity	Pancreatic cell line	(192)
	Herpes simplex Virus (HSV-1)	Increased T cell activation	Oral cancer	(193)
	Pox Virus	Enhance TILs selectivity by TME altering	Colon	(194)
	Adeno Virus Reo Virus Vaccinia Virus Herpes Simplex Virus	Choosing the best virus to increase the performance of TIL therapy	Solid tumors	(195)
**Cancer Vaccine**	Mutant Peptide	Increasing Survival Rate	Melanoma	(196)
	Mutant intracellular Protein	Durable Tumor Regression	Metastatic Melanoma	(197)
	Whole Tumor Lysate of Dc Vaccine	Increase Viability and Safety of Treatment	Metastatic Melanoma	(198)
	Matured Dc Vaccine in Presence of IL-12 & Toll Like Receptor Agonists	Allogenic T Cell Activation Correlated With IL-12 Production	Melanoma	(199)

NA. Not available.

Different forms of therapies, including conventional chemo/radiotherapy, cytokine therapy (IL-2, IL-15, IL-12, GM-CSF, TNF-α, IFN-α, IFN-γ), ICIs (antibodies against PD-1, PD-L1, TIM-3, OX40, CTLA-4, LAG3), vaccines (DC-based vaccines, neoadjuvant) and chemokines (CXCR2, CXCR4) in combination with TILs could be effective strategies to improve TIL infiltration and function in solid tumors (200). Furthermore, by infecting tumor cells with the virus, oncolytic virotherapy causes the emergence of new tumor antigens and creates local signals for optimal activation of T cells and polarization of M2 macrophages in the TME (193). These combination therapies enhance TIL therapy’s effectiveness and require more clinical trials.

### 6.1 TILs and chemo/radiotherapy

Chemotherapy has been accepted as a standard conventional method for treating all cancers. However, various adverse effects, poor bioavailability, high-dose requirements, low therapeutic indexes, multiple drug resistance, and non-specific targeting have limited the effectiveness of chemotherapy (201). Additionally, several experimental studies have demonstrated that the immunogenicity of resistant tumor cells and the host’s immune response are crucial factors in chemotherapy’s efficacy (202). Therefore, combining chemotherapeutics with immunotherapy is a promising approach for improving the clinical outcomes of cancer patients.

Study on patients with epithelial ovarian cancer, scientists compared the effects of the adoptive transfer of TILs alone with those of TILS therapy and a cisplatin-containing chemotherapeutic regimen. They found that patients’ complete response rate (CRR) was 30% and 70%, respectively. Interestingly, the second group showed no relapse after 15 months of monitoring (22). In another clinical study of osteosarcoma, patients with poor responses to chemotherapy were treated *via* chemotherapy plus TILs therapy (189). This study showed that the rate of DFS and overall survival (OS) were significantly increased in those who received TILs plus chemotherapy. Moreover, no significant TIL-related adverse effects were seen in the group treated with TIL plus chemotherapy. Therefore, combining TIL and chemotherapy may increase the survival of patients and reduce the defects of chemotherapy.

Although the clinical outcomes of combining radiotherapy and TIL therapy have not yet been reported, there is evidence that radiotherapy enhances the number of TILs recruited into the TME, boosting the anti-tumor response and prolonging patient survival. For instance, a study on head and neck cancer observed that the frequency of infiltrated TILs into the tumor site increased following radiotherapy and chemo/radiotherapy. Furthermore, this investigation reported that the expression of HIF-α as a hypoxia indicator decreased following the combination therapy (203).

Another clinical trial investigated the relationship between TILs and the effects of radiotherapy. The findings showed that radiotherapy causes an increase in the TILs frequency in patients with relapsed breast cancer (204). Another study compared the combination of chemo and radiotherapy with drug therapy in non-small cell lung cancer. The researchers observed that following chemo/radiotherapy, the expression of PD-L1 molecules in tumor cells and the frequency of CD8^+^ TILs were significantly increased (105). This study suggested that using PD-L1 monoclonal antibody can improve the effects of this treatment.

Collectively, it can be concluded that radiotherapy alone or in combination with chemotherapy or immune checkpoints causes an increase in the number of TILs. As a result, it changes the immunosuppressive space to the antitumoral space and, consequently, increases the survival of patients.

### 6.2 TILs and immune checkpoint inhibitors

The immunosuppressive nature of the TME caused by its components, including Tregs, M2 macrophages, and MDSCs, leads to the inactivation of TILs *in vivo* (205). Additionally, co-inhibitory molecules and signals in the TME promote angiogenesis and suppress antitumor T cell responses (113). Activated T cells express multiple co-inhibitory receptors, including LAG3, B and T lymphocyte attenuator (BTLA), CTLA-4, and PD- 1 (113). On the other hand, MDSCs and Tregs, through producing molecules such as TGF-β, IL-10, IL-35, and IDO, can promote the expression of co-inhibitory molecules on TILs (1, 113). Therefore, the blockade of co-inhibitory immune checkpoints could be an effective strategy to improve TIL infiltration and function.

Anti-PD1 is a frequent ICI combined with TIL therapy. Patients with metastatic cervical cancer with low microsatellite instability and negative PD-L1 were examined for anti-PD1 plus TIL therapy (14). Their findings demonstrated that the prognosis for metastatic cervical cancer is greatly improved by combining TILs and anti-PD1. It has already been shown that increased expression of PD-L1 on tumor and immune cells. The high microsatellite instability is associated with favorable responses to immunotherapy (206). In another study, researchers investigated whether TILs plus anti-PD1 improve the prognosis of patients with chemotherapy-resistant metastatic osteosarcoma. They reported that combined therapy was safe and improved the efficacy of TIL therapy. Besides, all treatment-related adverse events were reversible or manageable (190).

A study showed that combining TILs with ipilimumab in metastatic melanoma improved antitumor responses and increased survival time (191). In a phase I/II clinical trial, the co-administration of ipilimumab and nivolumab in combination with TIL therapy in different solid tumors was studied. Ipilimumab was administered before tumor resection, and nivolumab was used along with TIL infusion. Preconditioning chemotherapy was given before TIL infusion, followed by a low-dose stimulation with IL-2. They showed that adding ICIs before and during TIL infusion with low-dose IL-2 resulted in manageable toxicity and sizeable tumor regressions (51).

Taken together, TIL therapy combined with ICIs is more beneficial than monotherapy, leading to lower toxicity, improved prognosis, increased survival rate, and enhanced antitumor responses.

### 6.3 TILs and oncolytic virotherapy

Oncolytic viruses induce immunogenic cell death and indirectly increase T cells’ effectiveness through manipulating the immunosuppressive condition of the TME, releasing TAAs, inflammatory cytokines, and chemokine. In addition, oncolytic viruses directly lysis and destroy tumor cells (207, 208). In a unique design, Haminki et al. investigated the dual administration of TIL and virus in immunocompromised animals to induce virus-mediated tumor lysis along with enhanced TIL infiltration. They showed increased infiltration of TILs coupled with tumor regression and enhanced antitumor activity following combination therapy compared to the controls (192).

In another study, herpes simplex virus 1 (HSV-1), an oncolytic virus, was engineered to express OX40L and IL-12 (OV-OX40L/IL12). Infection of tumor cells with the engineered virus provided activation signals for T cells. Co-culture of the virus-infected cells with TILs, upregulation of MHC I, MHC II, and costimulatory receptors, such as CD80 and CD86 on tumor cells and increased the IFN-γ-secreting cells, leading to delayed tumor growth and improved survival time (193). In another approach, local administration of oncolytic poxvirus enhanced the activity of tumor-specific TILs by altering the immunosuppressive TME to an immunogenic milieu, leading to a significant reduction in tumor size and enhanced survival in mice harboring MC38 tumors (194). Determining the ideal oncolytic virus in each tumor type is necessary, and efforts to achieve this goal are ongoing. Studies on mouse models of tumors suggest that adenovirus might be the most effective virus for enhancing TIL therapy. Adenovirus, vaccinia virus, HSV, and reovirus are four oncolytic viruses currently being evaluated in clinical trials. Focus on virus engineering by arming with transgenes can provide potent antitumor effects, especially in combinational therapies (195).

### 6.4 TILs and cancer vaccines

#### 6.4.1 Neoantigen-based therapeutic cancer vaccines

Cancer neoantigens, the product of chromosomal changes, have unique amino acid sequences capable of inducing a potent and long-lasting immunological response. A high mutational and neoantigen burden in melanoma patients receiving TIL is substantially linked to increased PFS and OS (196). For patients with advanced solid tumors, therapeutic targeting of neoantigens using either ACT or vaccines has shown some early promise (209). At the National Cancer Institute, scientists have developed an approach to identify applicable immunogenic mutations by directly presentation of the tumor’s mutation profile to the patient’s APC, creating a renewable target for testing TILs’ reactivity. They showed immune recognition of the mutated intracellular proteins by patients’ TILs in metastatic melanoma (197). Taken together, the combination of genomics and cellular immunotherapy permits the identification of somatic alterations and the prediction of potential neoantigens that could be utilized as targets in cancer vaccines and ACT with TILs or engineered T cells (210).

#### 6.4.2 Dendritic cell-based therapeutic cancer vaccines

DCs are professional APCs that can be employed as powerful inducers of tumor-specific immune responses in cancer vaccines. Monocytes can be used to generate DCs *in vitro*, which can then be transfected with RNA encoding tumor-specific epitopes or pulsed with proteins, peptides, or whole-tumor lysates (211). There is a justification for mixing ACT and DC vaccines. Poschke et al. showed the viability and safety of this treatment strategy in a pilot phase I clinical study combining whole-tumor lysate DC vaccination with TILs in eight recruited metastatic melanoma patients (212).

An improved protocol for producing DC vaccines could induce more robust IL-12 production and T cell activation (198). Five patients received TIL therapy alone in an initial cohort to evaluate vaccine safety and optimize TIL expansion protocol. Five other patients received TILs combined with autologous tumor lysate-loaded DC vaccine in the second cohort. All patients received cyclophosphamide/fludarabine preconditioning and intravenous IL-2 after TIL transfer. In the safety/optimization cohort, all patients had a mixed response or stable disease, but none were durable. In the combination cohort, some patients experienced complete responses while others had partial responses.

In summary, they reported clinical responses by TIL therapy combined with DC vaccination in all treated metastatic melanoma patients who previously failed with ICIs (199). Using a modified DC vaccine, the authors investigated the efficacy of combined DC vaccination with CD40 agonistic antibodies in immune-competent mice with PDAC (213). Mice were vaccinated with syngeneic bone marrow-derived DCs loaded with either pancreatic cancer (in KPC mice) or mesothelioma (in AE17 mice) lysate and consequently treated with FGK45 as a CD40 agonist. Mesothelioma-lysate-loaded DCs combined with CD40 agonist-induced tumor growth reduction and improved survival time rather than anti-CD40 alone.

Together, combination therapies with TIL are more successful than TIL monotherapy in decreasing tumor development, improving patients’ clinical conditions and survival, and lowering adverse effects.

## 7 Next-generation TIL therapy

Although clinical trials have shown that polyclonal tumor-reactive T cells can mediate antitumor responses and are effective in patients with metastatic tumors, most patients did not experience a successful outcome (214).

Several key factors influence treatment efficacy with polyclonal TILs. TIL phenotype profoundly affects the efficacy of the anticancer response. Pre-selection of CD39- CD69- T cells exhibiting a stem-like phenotype with self-renewal and proliferation capacities, resulting in effective antitumor responses compared to terminally differentiated T cells (215). Therefore, a potential approach that promotes the stemness phenotype could enhance the antitumor potency. Also, inhibition of metabolism/anabolism, such as glycolysis and amino acid synthesis and acquisition, and blocking the signaling cascades that promote cell differentiation and growth, such as the PI3K/AKT/mTOR and MAPK pathways, may enhance T cell stemness and antitumor potency (216, 217). Another approach that enhances the survival and performance of traditional TIL therapy in cancer patients, when T cells are already highly differentiated with lacking stemness potential, is next-generation strategies (214). Such strategies include gene-editing technologies such as CRISPR and transcription-activator-like effector nuclease (TALEN) to genetically transfer and permanently modify the polyclonal TILs, to overexpress an interesting gene by viral transduction or knockout of the target gene (164). PDCD1 gene knockout of the TILs using CRISPR-Cas9 gene editing and TALEN technology prevents its binding to PD-L1 in the TME and increases TILs functionality (163). PD-1 knockout in TILs is also an alternative to combining TIL-based therapy with systemic ICIs, significantly reducing the unwanted side effects and toxicity associated with systemic ICIs (218). Another CRISPR approach that can be exploited to enhance T cell effector function is using CRISPR/Cas9 gene editing to abolish the expression of GATA3 transcription factors. GATA3 is highly expressed in CD8^+^ TIM3^+^ TILs and is involved in T cell dysfunction and inhibition of IFN-γ and IL-2 production upon stimulation (219). Also, CRISPR-mediated deletion of TILs cytokine-induced SH2 (CISH) gene leads to favorable outcomes and actively inhibits TCR signaling in CD8^+^ T cells (220).

In the clinic, the main focus of next-generation TIL is on engineering TIL to overexpress cytokines such as IL-2 and IL-12. T cell genetic modifications to secrete cytokines or express tethered cytokines can increase the antitumor activity and the lifespan of TILs by maintaining high cytokine levels preferentially at the tumor site. In addition, avoiding the systemic side effects of IL-2 administration during TIL treatment is necessary for T cells’ survival. TILs transduced with the gene encoding recombinant IL-2 showed promising results *in vitro*. Six of eight transduced patient samples produced IL-2 upon autologous tumor stimulation and survived longer than non-transduced TILs. Unfortunately, in clinical trials, poor *in vivo* responses were inconsistent with *in vitro* findings (221). IL-12 is a vital cytokine in perpetuating Th1 antitumor responses. TIL transduced with IL-12 under the regulation of nuclear factor of activated T cells (NFAT)-inducible promoter revealed favorable clinical effects in a phase I trial (222). Another manipulation that improves TIL therapy and is currently in clinical trials at *MD Anderson Cancer Center* (NCT01740557) is CXCR2 retrovirally transduced TILs. This TIL chemotactically localizes the tumor and ensures that the infused cells are localized to the tumor sites (223).

## 8 Concluding remarks and future directions

The high diversity of TCR, excellent ability to infiltrate into the tumor site, and low toxicity of TILs are the advantages of TIL therapy over other ACTs. TIL therapy is generally performed as a second-line treatment. The number of clinical trials on TIL therapy is increasing. Melanoma is still at the top of the number of TIL therapy clinical trials, followed by NSCLC, ovarian, and head and neck cancers. The success of two TIL products, LN144 and LN145, by Iovance in 2018 has paved the way for commercializing TIL therapy. However, TIL therapy still faces serious challenges. The most widespread method of TIL production is to isolate it from tumor tissue and then expand it *in vitro*. The process of selected TIL production usually takes 6-8 weeks. This long period causes TIL exhaustion. Besides, patients might be unable to wait for such a long time. Preparing young TILs without selection with antitumor reactivity is much faster than selecting the TILs. However, their tumor reactivity is questionable.

Additionally, the immunosuppressive mechanisms in the TME limit the TIL function. Also, injection of high-dose IL-2 as a standard method to support the growth and activity of injected TILs has several adverse effects. Combining TIL therapy with ICIs, modified cytokines (superkine), cancer vaccines, and next-generation TILs could minimize the limitations and maximize the efficacy of TIL therapy. Finally, developing multi-omics and sequencing techniques could help us set up a standard platform for rapidly expanding and selecting tumor-reactive TILs for each patient as personalized immunotherapy.

## Author contributions

MK. Conceived and designed the study, drafted the manuscript; MS. Contributed in manuscript drafting and critically evaluate the article; AN. Prepared **Table 2** and contributed in manuscript drafting; AR. Participated in the manuscript drafting and revision; ZB. Prepared **Table 1** and contributed in manuscript drafting; HK. Contributed in study design and manuscript drafting, prepared figures, revised the manuscript; RF. Designed and supervised the project, revised and finalized the manuscript; All authors contributed to the article and approved the submitted version.

## Funding

Iran University of Medical Sciences supported this study.

## Conflict of interest

The authors declare that the research was conducted in the absence of any commercial or financial relationships that could be construed as a potential conflict of interest.

## Publisher’s note

All claims expressed in this article are solely those of the authors and do not necessarily represent those of their affiliated organizations, or those of the publisher, the editors and the reviewers. Any product that may be evaluated in this article, or claim that may be made by its manufacturer, is not guaranteed or endorsed by the publisher.
